# Simvastatin re-sensitizes hepatocellular carcinoma cells to sorafenib by inhibiting HIF-1α/PPAR-γ/PKM2-mediated glycolysis

**DOI:** 10.1186/s13046-020-1528-x

**Published:** 2020-01-30

**Authors:** Jiao Feng, Weiqi Dai, Yuqing Mao, Liwei Wu, Jingjing Li, Kan Chen, Qiang Yu, Rui Kong, Sainan Li, Jie Zhang, Jie Ji, Jianye Wu, Wenhui Mo, Xuanfu Xu, Chuanyong Guo

**Affiliations:** 1 Department of Gastroenterology, Shanghai Tenth People’s Hospital, Tongji University School of Medicine, NO. 301, Middle Yanchang Road, Jing’an District, Shanghai, 200072 China; 2 Department of Gastroenterology, Putuo People’s Hospital, Tongji University School of Medicine, Shanghai, 200060 China; 3 Department of Gastroenterology, Zhongshan Hospital of Fudan University, Shanghai, 200032 China; 4 Shanghai Institute of Liver Diseases, Zhongshan Hospital of Fudan University, Shanghai, 200032 China; 5 Shanghai Tongren Hospital, Shanghai Jiaotong University School of Medicine, Shanghai, 200336 China; 6 Department of Gastroenterology, Shanghai General Hospital, Shanghai Jiaotong University School of Medicine, Shanghai, 200080 China; 7 Shanghai Tenth Hospital, School of Clinical Medicine of Nanjing Medical University, Shanghai, 200072 China; 8 Department of Gastroenterology, Shidong Hospital of Shanghai, Shanghai, 200433 China

**Keywords:** Sorafenib resistance, Simvastatin, Glycolysis, Hepatocellular carcinoma, PKM2, HIF-1α, PPAR-γ

## Abstract

**Background:**

Hepatocellular carcinoma (HCC) is a common primary malignant tumor which usually progresses to an advanced stage because of late diagnosis. Sorafenib (Sora) is a first line medicine for advanced stage HCC; however, it has been faced with enormous resistance. Simvastatin (Sim) is a cholesterol-lowering drug and has been reported to inhibit tumor growth. The present study aims to determine whether Sora and Sim co-treatment can improve Sora resistance in HCC.

**Methods:**

The HCC cell line LM3 and an established Sora-resistant LM3 cell line (LM3-SR) were used to study the relationship between Sora resistance and aerobic glycolysis. Cell proliferation, apoptosis and glycolysis levels were analyzed by western blotting, flow cytometry analysis and biomedical tests. A xenograft model was also used to examine the effect of Sim in vivo. Detailed mechanistic studies were also undertaken by the use of activators and inhibitors, and lentivirus transfections.

**Results:**

Our results demonstrated that the resistance to Sora was associated with enhanced aerobic glycolysis levels. Furthermore, LM3-SR cells were more sensitive to Sim than LM3 cells, suggesting that combined treatment with both Sora and Sim could enhance the sensitivity of LM3-SR cells to Sora. This finding may be due to the suppression of the HIF-1α/PPAR-γ/PKM2 axis.

**Conclusions:**

Simvastatin can inhibit the HIF-1α/PPAR-γ/PKM2 axis, by suppressing PKM2-mediated glycolysis, resulting in decreased proliferation and increased apoptosis in HCC cells, and re-sensitizing HCC cells to Sora.

## Background

Hepatocellular carcinoma (HCC) is one of the most common primary malignant tumors worldwide and according to global cancer statistics (2018), liver cancer is the fourth leading cause of cancer deaths [1–3]. The pathogenesis of HCC is associated with chronic viral hepatitis infection, alcohol abuse, and aflatoxin B1 intake [4]. The standard therapeutic methods for the treatment of HCC include surgical resection, trans-arterial embolization, radiotherapy and chemotherapy. However, these treatments are often inadequate because of delays in diagnosis and metastasis, resulting in advanced HCC [5].

Sorafenib (Sora) is a multikinase inhibitor and has been approved as the first-line targeted therapy for advanced HCC [2, 6]. In two phase III trials, results showed that Sora could prolong the overall survival of HCC patients by 2–3 months. However, only 30% of patients benefitted from Sora because of acquired resistance which occurred within 6 months [7, 8]. The mechanisms of Sora resistant are complex and undefined, but include increased epidermal growth factor receptor (EGFR) expression, c-Jun and Akt activation of HCC cells, epithelial-mesenchymal transition (EMT), increased cancer stem cells, and an increase in the hypoxic environment [2, 5]. Recently, researchers have found that Sora can impair oxidative phosphorylation (OXPHOS) and promote aerobic glycolysis in HCC [9, 10]. Although aerobic glycolysis is a hallmark of cancer, few studies have attempted to elucidate the relationship between aerobic glycolysis and Sora resistance.

Currently, strategies to prevent Sora resistance include co-treatment with other medicines in clinical use, including agents that target specific molecules such as anti-EGFR antibodies, (Cetuximab), cytotoxic chemotherapeutic drugs (Epirubicin, Cisplatin, 5-FU and Capecitabine), and immunotherapeutic drugs (anti-PD-1 antibodies) [2, 11, 12]. However, this combinational therapy is usually limited due to severe adverse side-effects, such as diarrhea, and organ damage [11]. Therefore, a safer agent is needed to overcome or improve Sora resistance in HCC.

Simvastatin is a cholesterol-lowering statin, which can inhibit the activity of hydroxymethylglutaryl coenzyme A (HMG CoA) reductase and prevent the synthesis of cholesterol. Recently, many studies have demonstrated that statins also have additional benefits, including antioxidant, anti-proliferative, and anti-inflammatory and can function to protect the vascular endothelium [13–15]. Statins also play a role in the prevention of liver diseases, including non-alcoholic fatty liver disease, cholestatic liver disease, and liver cirrhosis [16, 17]. Moreover, statins always exhibit synergistic effects when combined with other chemotherapeutic drugs [18]. For example, fluvastatin has been reported to increase the cytotoxicity of Sora in melanoma cells [19]. Kim et al. found that co-treatment with lovastatin and enzastaurin, both PKC inhibitors, synergistically inhibited HCC cell growth in vitro and in vivo [20]. Some researchers have reported that the combination of celecoxib or NS 398 and statins enhanced the inhibition of HCC growth [21, 22]. However, there are few studies investigating the combined treatment of Sim and Sora to treat Sora-resistant-HCC, and the effect on aerobic glycolysis.

Therefore, in the present study, we combined Sora with Sim to determine a role for Sim in the treatment of Sora resistance in HCC, and to explore the potential mechanisms.

## Materials and methods

### Reagents

Sorafenib tosylate, FG-4592, BAY87–2243, Rogislitazone, GW9662, Compound 3 k and DASA 58 were purchased from Selleck Chemicals (Shanghai, China). Sim was purchased from Yuanye Biotechnology (Shanghai, China). The cell counting kit (CCK-8) and the nuclear and cytoplasmic protein extraction kit were obtained from Yeasen Biotechnology (Shanghai, China), the Hoechst 33342 fluorescence staining kit was from Servicebio (Wuhan, China), the Annexin V-FITC apoptosis assay kit was from BD Biosciences (San Jose, CA, USA), and the primary antibodies used in the study are listed in Table 1.

**Table 1:** The primary antibodies used in the study

Antibody	Species	Targeted species	Dilution ratio	Supplier	Catalogue number
β-actin	M	H, M, R	1:1000	CST	3700
PCNA	Rbt	H, M, R	1:2000	PT	10,205–2-AP
Bax	M	H, M, R	1:1000	PT	60,267–1-Ig
Bcl-2	Rbt	H	1:1000	CST	15,071
Caspase 3	Rbt	H, M, R	1:1000	PT	19,677–1-AP
HK2	Rbt	H, M, R	1:1000	PT	22,029–1-AP
PFKL (PFK1)	Rbt	H, M	1:1000	CST	8175
PKM2	M	H, M, R	1:1000	PT	60,268–1-Ig
OXPHOS	M	H, M, R	1:250	MT	MS604
HIF-1α	Rbt	H	1:1000	PT	20,960–1-AP
PPAR-γ	Rbt	H, M, R	1:1000	ABclonal	60,318–1-Ig
Cleaved PARP	Rbt	H	1:1000	CST	5625S
Cleaved PARP	M	M	1:1000	CST	9548S
Ki-67	Rbt	M, R	1:1000	Servicebio	GB111141
LaminA/C	Rbt	H, M, R	1:1000	PT	10,298–1-AP
β-Tubulin	Rbt	H, M, R	1:1000	PT	10,094–1-AP

Abbreviations for the table: *H* human; *M* mouse; *Rbt* rabbit; *R* rat; *CST* Cell Signaling Technology (Danvers, MA, USA). *PT* Proteintech (Chicago, IL, USA). ABclonal Biotechnology (Wuhan, China). *MS* Mitoscience (St. Louis Park, MN, USA)

### Cell culture

Four different HCC cell lines, including HCC-LM3, SMMC-7721, Bel-7402, and Huh-, a hepatoblastoma cell line HepG2 [23], and the LO2 normal human liver cell line were purchased from the Cell Bank of Type Culture Collection of the Chinese Academy of Sciences (Shanghai, China), and maintained in high glucose Dulbecco’s Modified Eagle Medium (DMEM HyClone, GE Healthcare, Logan, UT, USA) supplemented with 10% fetal bovine serum, 100 U/mL of penicillin, and 100 g/mL of streptomycin (all from Gibco, Thermo Fisher Scientific, Waltham, MA, USA).

### Establishment of SORA-resistant LM3 cells

The establishment of SORA-resistant LM3 cells (LM3-SR) was conducted according to previous studies [24, 25]. Briefly, LM3 cells were cultured in a step-wise increase in Sora concentration (4–10 μM), by 10% every two weeks until the maximum tolerated dose (10 μM) had been reached. LM3-SR cells were cultured in the presence of 1 μM Sora, which was withdrawal for three days before analysis.

### CCK8 assay, quantitative reverse transcription-polymerase chain reaction (qRT-PCR) and western blotting

The primers used in the study were synthesized by Generay Biotech (Shanghai, China), and their sequences listed in Table 2. The PrimeScript RT Reagent kit and SYBR Premix Ex Taq were purchased from TaKaRa Biotechnology (Dalian, China). CCK8 assay, quantitative RT-PCR (qRT-PCR), and western blotting were conducted as described previously [26–28]. The effects of different drugs were determined using CCK8 assay. Therefore, Sora at a concentration of 15 μM and Sim at 10 μM or 50 μM were used in the following studies where treatment was given for 24 h.

**Table 2:** Primers used for qPCR

Gene name	Forward (5′-3′)	Reverse (5′-3′)
PKM2	ATGTCGAAGCCCCATAGTGAA	TGGGTGGTGAATCAATGTCCA
HK2	GAGCCACCACTCACCCTACT	CCAGGCATTCGGCAATGTG
PFKFB1	AGAAGGGGCTCATCCATACCC	CTCTCGTCGATACTGGCCTAA
PFKFB2	TGGGCCTCCTACATGACCAA	CAGTTGAGGTAGCGTGTTAGTTT
PFKFB3	TTGGCGTCCCCACAAAAGT	AGTTGTAGGAGCTGTACTGCTT
PFKFB4	TCCCCACGGGAATTGACAC	GGGCACACCAATCCAGTTCA
LDH-A	ATGGCAACTCTAAAGGATCAGC	CCAACCCCAACAACTGTAATCT
LDH-B	TGGTATGGCGTGTGCTATCAG	TTGGCGGTCACAGAATAATCTTT
LDH-C	AGAACATGGTGATTCTAGTGTGC	ACAGTCCAATAGCCCAAGAGG
HIF-1α	GAACGTCGAAAAGAAAAGTCTCG	CCTTATCAAGATGCGAACTCACA
AMPK-α1	TTGAAACCTGAAAATGTCCTGCT	GGTGAGCCACAACTTGTTCTT
AMPK-α2	GTGAAGATCGGACACTACGTG	CTGCCACTTTATGGCCTGTTA
AMPK-β1	CCACTCCGAGGAAATCAAGGC	CTGGGCGGGAGCTTTATCA
GLUT1	GGCCAAGAGTGTGCTAAAGAA	ACAGCGTTGATGCCAGACAG
β-actin	CATGTACGTTGCTATCCAGGC	CTCCTTAATGTCACGCACGAT
PGC1	TCTGAGTCTGTATGGAGTGACAT	CCAAGTCGTTCACATCTAGTTCA
PPRC1	CAAGCGCCGTATGGGACTTT	GGAGGCATCCATGTAGCTCT
PPAR-α	ATGGTGGACACGGAAAGCC	CGATGGATTGCGAAATCTCTTGG
PPAR-γ	GGGATCAGCTCCGTGGATCT	TGCACTTTGGTACTCTTGAAGTT

### Standard colony formation, Hoechst 33342 staining, immunofluorescence staining and flow cytometry analysis for apoptosis

Standard colony formation, Hoechst 33342 staining, immunofluorescence staining and flow cytometry analysis for apoptosis were conducted as described previously [29]. The flow cytometry used in the study was FACSCalibur (Becton, Dickinson, Franklin Lakes, NJ, USA), and analyzed by FlowJo software (version 10; FlowJo LLC, Ashland, OR, USA). All the images were captured using Leica inverted fluorescence microscope DMI6000B (Leica Microsystems, Wetzlar, Germany).

### Biomedical analysis

Glycolysis levels were determined using the detection of lactate production and glucose uptake levels in LM3 or LM3-SR cells. The lactate assay kit was obtained from Nanjing Jiancheng Bioengineering Institute (Nanjing, China). Glucose uptake levels were calculated using a glucose detection kit from Rongsheng Biotechnology (Shanghai, China), and the values were normalized to the protein concentrations of the cell lysates [10, 30]. The triglyceride (TG), total cholesterol (TCHO) low-density lipoprotein cholesterol (LDL-C) and the high-density lipoprotein cholesterol (HDL-C) assay kits were all purchased from Nanjing Jiancheng Bioengineering Institute (Nanjing, China). All the experiments were carried out according to the manufacturer’s instructions.

### Plasmid construction, lentivirus packaging, and cell transfection

The lentivirus overexpression or knock down construct for PKM2 was synthesized by BioLink Biotechnology (Shanghai, China). All plasmids used in this study were verified by sequencing. Lentiviral transfection was conducted following the manufacturer’s instructions. The positive cells were selected by puromycin resistance and transfection efficiency was determined by qPCR and western blotting.

### Co-immunoprecipitation (co-IP) assay

The Co-IP assay was carried out using the Pierce Co-immunoprecipitation Kit (Thermo Scientific, Waltham, MA, USA) and according to manufacturer’s protocol, 1 mg of total protein lysate was mixed with 10 μg of primary antibody, or IgG. The results were analyzed by western blotting [29].

### Subcutaneous xenograft tumor model

Four-week-old nude mice were obtained from Shanghai Slack Laboratory Animal Co. LTD (Shanghai, China), and housed in a standard laboratory environment with free access to water and food. The study was approved by the Animal Care and Use Committee of Shanghai Tongji University, and conducted following the ARRIVE guidelines.

For the establishment of the subcutaneous xenograft tumor model, LM3 or LM3-SR cells were resuspended to a density of 3 × 10^6^/mL in serum-free DMEM and each mouse injected with 200 μL of cells in the upper flank region. Tumor volume was calculated as: volume (mm^3^) = 0.5 × (major axis) × (minor axis)^2^, and when it reached 100 mm^3^, mice received Sora (10 mg/kg), Sim (10 mg/kg) or a co-treatment with Sora (10 mg/kg) + Sim (10 mg/kg) orally once daily until the end of the study. At day 28 post cell injection, mice were anesthetized with 1.25% pentobarbital, and blood samples were collected by removing the eyes, and the tumors, as well as heart, kidney and lung were resected and immersed in 4% paraformaldehyde.

The following study groups were used: (1) for the analysis of the LM3-SR cell-induced xenograft, 12 mice were randomly divided into four groups (*n* = 3): 1) LM3-SR-Control (CTRL) group, mice were injected with LM3-SR cells in the absence of Sora; 2) LM3-SR-Sora group, mice were injected with LM3-SR cells and received Sora (10 mg/kg) administration; 3) LM3-Control (CTRL) group, mice were injected with LM3 cells but with no Sora administration; 4) LM3-Sora group, mice were injected with LM3 cells and received Sora (10 mg/kg) administration. (2) For the analysis of Sora and Sim co-treatment, 16 mice were randomly divided into four groups (*n* = 4): 1) CTRL group, mice were injected with LM3-SR cells alone; 2) Sora group, mice were injected with LM3-SR cells and received Sora (10 mg/kg) administration; 3) Sim group, mice were injected with LM3-SR cells and received Sim (10 mg/kg) administration; 4) Sora + Sim group, mice were injected with LM3-SR cells and received both Sora (10 mg/kg) + Sim (10 mg/kg).

### Hematoxylin and eosin (H&E) staining, immunohistochemistry (IHC) staining, and terminal deoxynucleotidyl transferase dUTP nick end labeling (TUNEL)

Animal tissues were immersed in 4% paraformaldehyde for at least 24 h, then embedded in paraffin and cut into 3 μm thick sections. The H&E, IHC and TUNEL staining was carried out as described previously [31, 32].

### Statistical analysis

Data were presented as mean ± standard deviation (SD) from three independent experiments. After the statistical analysis was carried out the results were imaged using GraphPad Prism 6 software (GraphPad Software, San Diego, CA, USA). The comparisons between two groups were analyzed by Student’s t-tests (unpaired, two-tailed) or the one-way analysis of variance (ANOVA) (followed by Tukey’s post-hoc tests). *P* < 0.05 was considered statistically significant. All data generated and analyzed during this study are available on request from the corresponding author.

## Results

### The establishment and characteristics of Sora-resistant cells

**Fig. 1:**
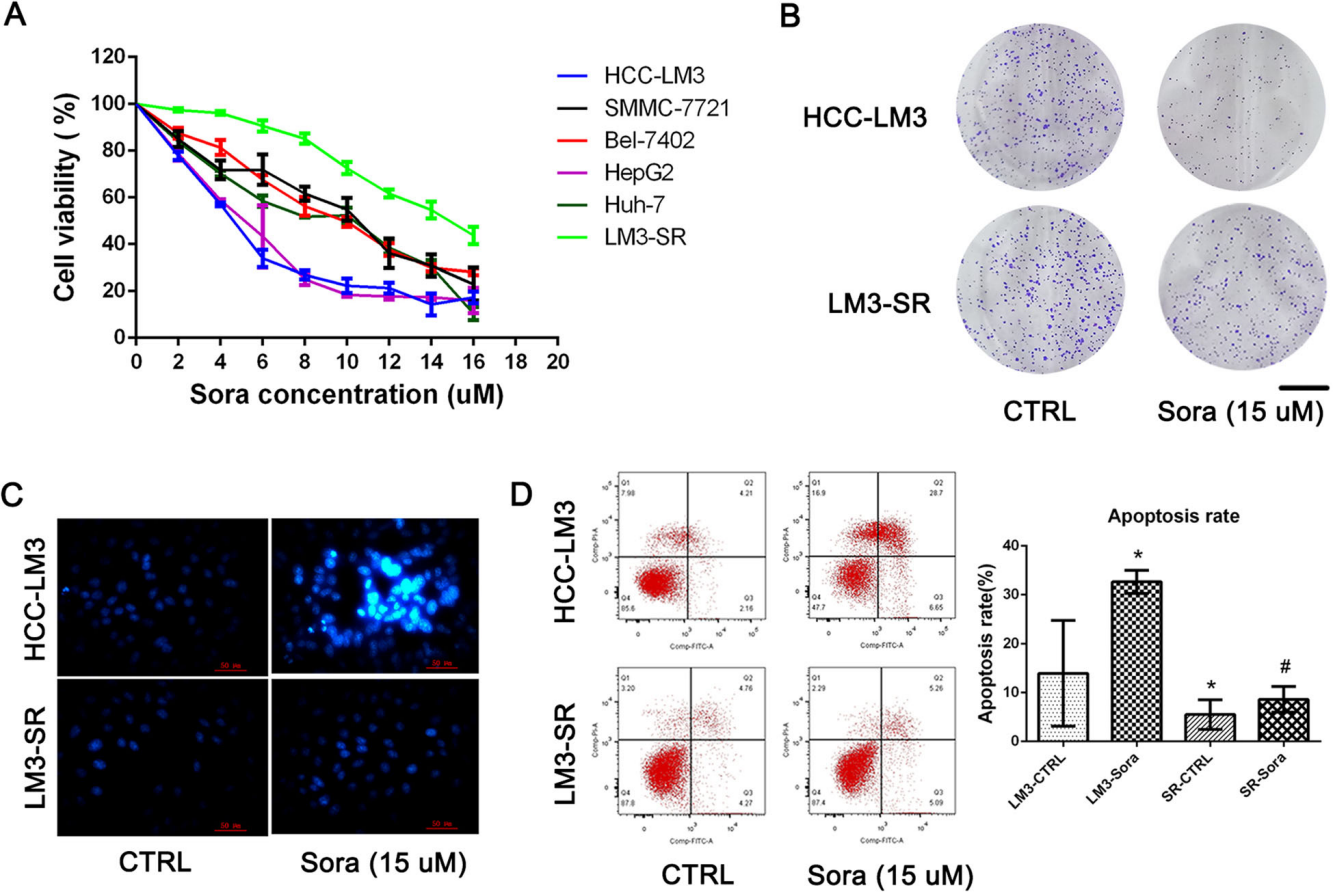
The characteristics of Sora-resistant cells. (**a**) The IC50 of five HCC cell lines and LM3-SR cells to Sora were calculated using the CCK8 assay. (**b**) The colony forming ability of LM3 and LM3-SR cells (scale bar, 1 cm). (**c**) Hoechst 33342 staining used to show bright apoptotic cells. (**d**) Flow cytometry of cells stained with FITC and PI to determine apoptotic cells. The data is represented as mean ± SD. * indicates *p* < 0.05 vs. LM3-CTRL group. # indicates *p* < 0.05 vs. LM3-SR-CTRL group

Both the primary and the secondary resistance to Sora have restricted the application and treatment effects of this drug in the clinic. Therefore, four types of HCC cell lines, including HCC-LM3 (shorted to LM3), SMMC-7721, Bel-7402 and Huh-7, and a hepatoblastoma cell line HepG2 were used to detect the half inhibitory concentration (IC50) of Sora in this study. As the results show in Fig. 1a, different concentrations of Sora were given for 24 h, and the cell viability was calculated using the CCK8 assay. The IC50s for Sora in these cell lines were 4.47 μM (LM3), 8.79 μM (SMMC-7721), 8.98 μM (Bel-7402), 4.65 μM (HepG2), and 7.26 μM (Huh-7), respectively. Since the IC50 of LM3 was the lowest among the five liver cancer cell lines, which meant that LM3 was the most sensitive to Sora. Therefore, LM3 cells were chosen as Sora-sensitive cells, and we then established the Sora-resistant LM3 cells line (LM3-SR) as control to explore the underlying mechanism in Sora resistance [9, 33, 34]. The IC50 of LM3-SR was 16.33 μM (Fig. 1a), which is approximately four-fold higher than LM3 cells. This implied the successful establishment of the Sora-resistant LM3 cells. Therefore, the LM3 and LM3-SR cells were used in the following study to represent the Sora-sensitive and Sora-resistant cells, at a Sora concentration of 15 μM.

The different characteristics seen between LM3 and LM3-SR cells were detected using proliferation assays and flow cytometry. As shown in Fig. 1b, 15 μM Sora clearly prevented colony formation in LM3 cells and this effect was reduced in LM3-SR cells, indicating that the ability to proliferate in LM3-SR cells was higher than in LM3 cells. Hoechst 33342 staining revealed that there were more positive cells (representing apoptotic cells) in the Sora treated group of LM3 cells, when compared to LM3-SR cells (Fig. 1c). Furthermore, flow cytometry (Fig. 1d) demonstrated that the apoptosis rate in LM3-SR cells when exposed to Sora was much lower than that seen in LM3 cells (32.65 ± 2.37% vs. 8.63 ± 2.62%). These findings suggest that it was difficult for Sora (15 μM) to induce apoptosis in LM3-SR cells. These results suggest that LM3-SR cells were resistant to Sora and were less likely to experience inhibition of proliferation or apoptosis.

### Resistance to Sora is associated with enhanced aerobic glycolysis

**Fig. 2:**
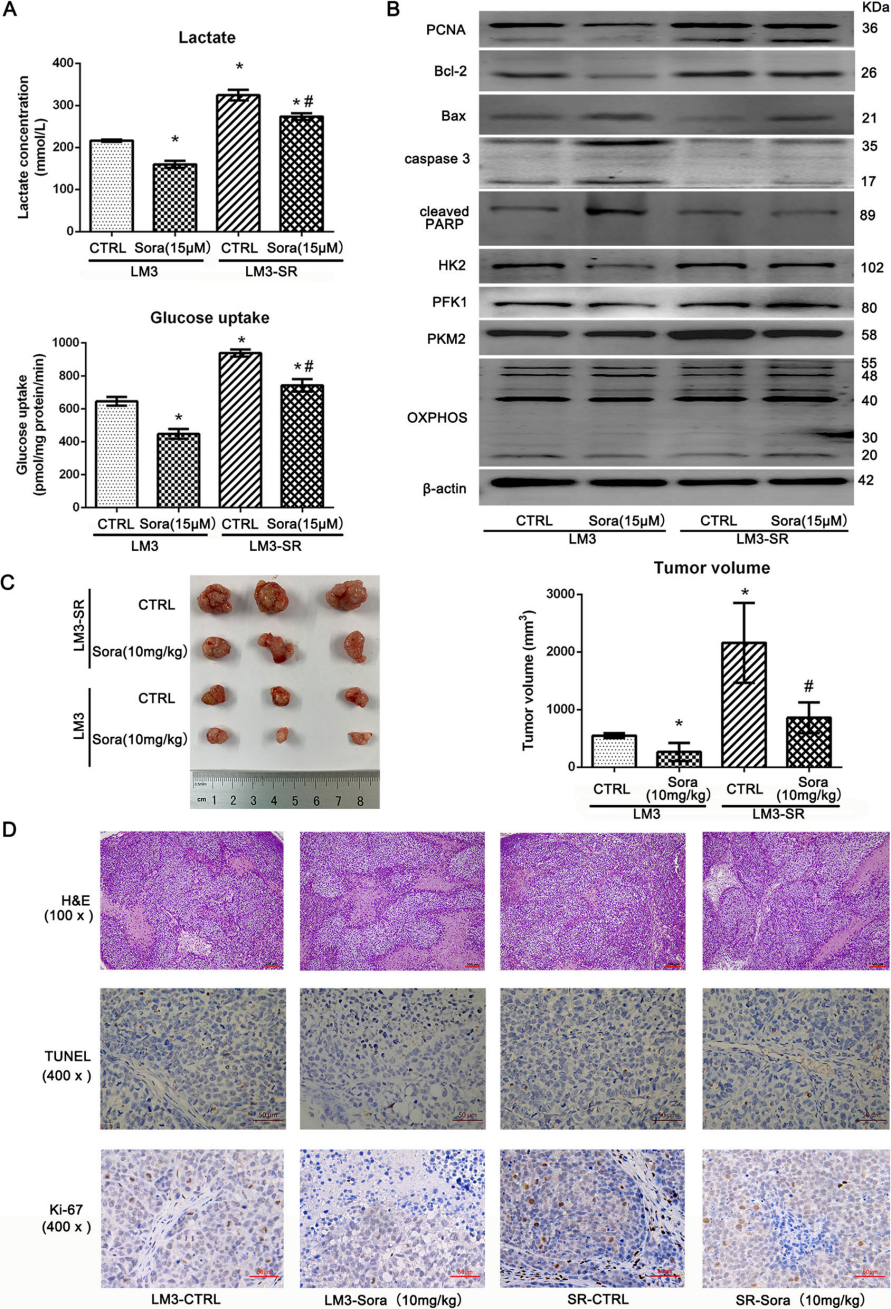
The resistance to Sora was associated with enhanced aerobic glycolysis in vivo and in vitro. (**a**) Glycolysis levels in LM3 and LM3-SR cells were detected by lactate production and glucose uptake levels. Both the glucose uptake and lactate production levels were higher in LM3-SR cells when compared to LM3 cells, and Sora (15 μM) decreased these effects in LM3-SR cells. (**b**) Western blotting analysis of three key enzymes during glycolysis, the proliferation indicator PCNA, and apoptosis markers Bcl-2, Bax, Caspase 3 and cleaved PARP. (**c**) Xenograft tumor model exhibiting tumor volume induced by LM3 and LM3-SR cells. LM3-SR cells could induce larger tumor volume than LM3 cells, and Sora (10 mg/kg) was unable to suppress tumor volume in the LM3-SR group but did so in the LM3 group (*n* = 3). (**d**) H&E, TUNEL and Ki-67 staining of tumors. There was less necrosis (pink areas) and apoptosis (the nucleus was stained with dark-brown), but more tumor parenchyma and higher Ki-67 staining in the LM3-SR group. The data is represented as mean ± SD. * indicates *p* < 0.05 vs. LM3-CTRL group. # indicates *p* < 0.05 vs. LM3-SR-CTRL group

Aerobic glycolysis is a hallmark of tumor cell metabolism, and enhanced aerobic glycolysis is characterized by high glucose uptake and high lactate production. Some studies have reported that long-term use of Sora could lead to increased aerobic glycolysis, and this may be associated with Sora resistance [9]. Therefore, in our study, glycolysis levels of LM3-SR cells were measured. The results presented in Fig. 2a show that both glucose uptake and lactate production were higher in LM3-SR cells when compared to LM3 cells, suggesting that LM3-SR cells produced higher glycolysis levels. Moreover, Sora (15 μM) was effective at inhibiting both glucose uptake and lactate production in LM3 cells; whereas Sora (15 μM) produced a slight decrease in LM3-SR cells. The three key enzymes involved in glycolysis, including hexokinase 2 (HK2), phosphofructokinase 1(PFK1) and pyruvate kinase, type M2 (PKM2), and OXPHOS were determined by western blotting (Fig. 2b). The results were similar to those seen in Fig. 2a, demonstrating that LM3-SR cells had higher expression levels of glycolysis-associated proteins and impaired OXPHOS associated proteins. The proliferation indicator PCNA, as wells as the apoptosis markers Bcl-2, Bax, caspase 3 and cleaved PARP were also detected, and the results demonstrated that Sora (15 μM) had a limited effect on inhibiting proliferation, glycolysis, and induction of apoptosis in LM3-SR cells. These findings indicate that Sora resistance was associated with enhanced glycolysis. Since Bel-7402 maybe a naïve Sora-resistant HCC cell line when compared to LM3, the mechanism underlying Sora resistance was also explored in Bel-7402 cells (Additional file 1: Figure S1).

To further explore the Sora-resistant effect in HCC cells in vivo, both LM3 and LM3-SR cells were seeded into nude mice to produce a xenografted tumor model (Fig. 2c). The results showed that LM3-SR cells could induce a larger tumor volume than LM3 cells, and Sora (10 mg/kg) was unable to suppress tumor volume in the LM3-SR groups as well as in the LM3 groups. The H&E, TUNEL and IHC staining of Ki-67 results showed that there were less necrotic (pink areas) and apoptotic areas (the nucleus was stained dark-brown), but more tumor parenchyma and higher Ki-67 staining in the LM3-SR groups (Fig. 2d), as well as a reduced treatment effect of Sora (10 mg/kg). In conclusion, these results show that the resistance to Sora in LM3-SR cells is associated with enhanced aerobic glycolysis levels, and this may result in the decreased effect of treatment seen in vivo.

### LM3-SR cells have a greater sensitivity to Sim when compared to LM3 cells

**Fig. 3:**
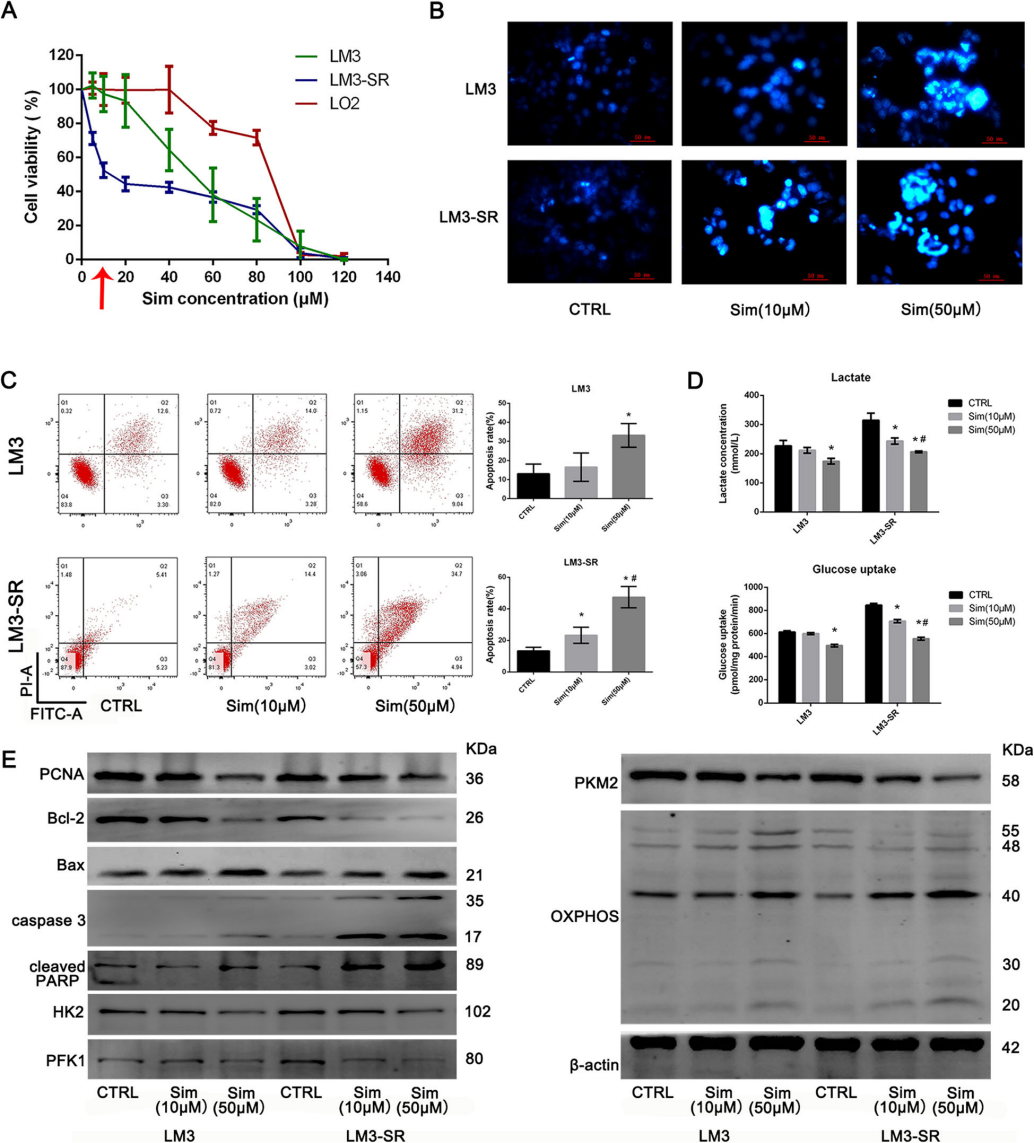
Effect of Sim on LM3 and LM3-SR cells. (**a**) CCK8 assay showed the effect of Sim on LM3, LM3-SR and normal liver cells (LO2). (**b**) Hoechst 33342 staining showing the effect of different concentrations of Sim on LM3 and LM3-SR cells. (**c**) Flow cytometry showing of effect of different concentrations of Sim on LM3 and LM3-SR cells. The data were represented as mean ± SD. * indicates *p* < 0.05 vs. CTRL group. # indicates *p* < 0.05 vs. Sim (10 μM) group. (**d**) Glycolysis levels after Sim treatment in LM3 and LM3-SR cells, reflected by lactate production and glucose uptake levels. (**e**) Western blotting analysis of critical proteins. The data were represented as mean ± SD. * indicates *p* < 0.05 vs. LM3-CTRL group. # indicates *p* < 0.05 vs. LM3-SR-CTRL group

Sim is a commonly used drug to lower blood lipid levels. Recently, anti-fibrotic and anti-cancer effects of Sim have been found. In our study, the effect of Sim on LM3, LM3-SR and normal liver cells LO2 were determined using CCK8 assay (Fig. 3a). Surprisingly, the IC50 of Sim in these cells were 55.99 μM (LM3), 14.93 μM (LM3-SR), and 74.92 μM (LO2) respectively, indicating that LM3-SR cells were 3.75 fold more sensitive to Sim than LM3 and LO2. Furthermore, Sim (10 μM) was able to kill LM3-SR cells without influencing the viability of normal liver cells (LO2). Therefore, two concentrations of Sim (10 μM and 50 μM) were used to explore their effects on apoptosis and glycolysis in LM3 and LM3-SR cells. Consistently, Sim (10 μM) did not significantly induced LM3 cell apoptosis, but apoptosis was higher in LM3-SR cells and the high dose of Sim (50 μM) was able to induce apoptosis in both LM3 and LM3-SR cells (Fig. 3b-c). The results in Fig. 3d show that Sim (10 μM) failed to decrease lactate production or glucose uptake in LM3 cells, whereas Sim (50 μM) was able to produce both affects. The two concentrations of Sim (10 μM and 50 μM) were able to reduce lactate production and glucose uptake in LM3-SR cells. Western blotting for PCNA, Bcl-2, Bax, Caspase 3, cleaved PARP, glycolysis-related enzymes and OXPOHS further verified the effect of Sim (Fig. 3e). Therefore, these results demonstrated that LM3-SR cells are more sensitive to Sim than LM3 cells, and Sim (10 μM) was effective at reducing proliferation, glycolysis and inducing apoptosis in LM3-SR cells.

### Sim enhanced the sensitivity of LM3-SR cells to Sora when used in combination

**Fig. 4:**
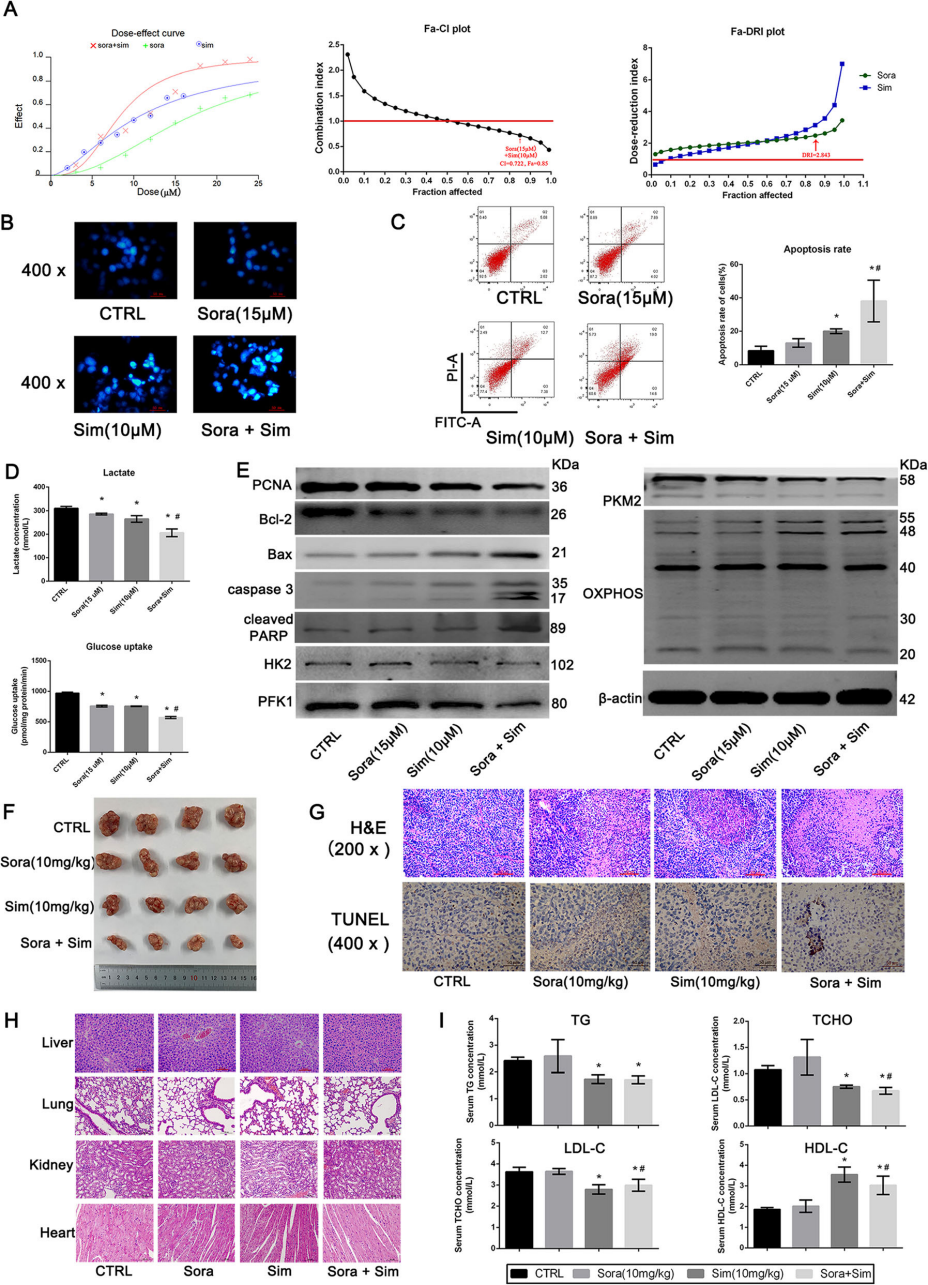
Combined treatment of Sora and Sim enhanced the sensitivity of LM3-SR cells to Sora. (**a**) The combined treatment analysis of Sora and Sim using Calcusyn. The dose-effect curve, Fa-CI plot and Fa-DRI plots are shown. Sora (15 μM) and Sim (10 μM) resulted in CI value of 0.722, and the DRI for Sora was 2.843. (**b**) Hoechst 33342 staining of Sora and Sim co-treatment in LM3-SR cells. (**c**) Flow cytometry analysis of the effect of Sora and Sim co-treatment in LM3-SR cells. (**d**) Glycolysis levels of Sora and Sim co-treatment in LM3-SR cells, reflected by lactate production and glucose uptake levels. (**e**) Western blotting analysis of critical proteins. (**f**) Effect of Sora and Sim co-treatment induced by LM3-SR cells using the xenograft tumor model. (**g**) H&E and TUNEL staining of tumor slices. (**h**) H&E staining of liver, kidney, lung and heart after Sim treatment (200 x). (**i**) Serum lipid levels, including TG, TCHO, LDL-C and HDL-C (*n* = 4). The data were represented as mean ± SD. * indicates *p* < 0.05 vs. CTRL group. # indicates *p* < 0.05 vs. Sora group

Since LM3-SR cells were more sensitive to Sim, we combined Sora (15 μM) with Sim (10 μM, IC50 1:1 combination) to determine whether Sim can enhance the sensitivity of LM3-SR cells to Sora. In Fig. 4a, different doses of Sora and Sim were combined at a constant ratio, and the effects analyzed by CKK8 assay. The fraction affected-combination index (Fa-CI) plot shows that the CI calculated for Sora (15 μM) and Sim (10 μM) was 0.722 (CI < 1), indicating a synergistic effect. The dose reduction index (DRI) was also calculated, and we found that Sim (10 μM) was able to reduce the dose of Sora by 2.843-fold. The apoptosis assays also reflected that both Sora + Sim treatment enhanced the apoptosis rate of LM3-SR cells (Fig. 4b-c). Lactate production and glucose uptake levels with both Sora + Sim was also lower than Sora (15 μM) or Sim (10 μM) treatment alone (Fig. 4d), again inferring a synergic effect on the suppression of glycolysis by Sora + Sim co-treatment. Western blotting analysis of PCNA, Bcl-2, Bax, Caspase 3, cleaved PARP, glycolysis-related enzymes and OXPOHS further confirmed a synergistic effect of Sora + Sim co-treatment (Fig. 4e). In addition, we also explored the co-treatment of Sora (5 μM) and Sim (10 μM) in LM3 cells, and the synergic effects were also observed in LM3 cell (Additional file 2: Figure S2).

We also explored the co-treatment of Sora + Sim on HCC cells in vivo. As show in Fig. 4f, the tumor volume in the Sora + Sim treated group was much smaller than when Sora or Sim were treated alone. H&E and TUNEL staining demonstrated that there were more necrotic and apoptotic areas in the Sora + Sim group. Furthermore, we also examined the pathological manifestations of important organs to determine whether Sim was detrimental to organ function. The H&E staining seen in Fig. 4h demonstrated that Sim did not harm the liver, lungs, kidneys or heart. Since Sim was used as a lipid-lowering drug, serum lipid levels were also detected. The results in Fig. 4i revealed that Sim could lower TG, TCHO and LDL-C levels, but increased HDL-L levels in nude mice; whereas Sora + Sim co-treatment could also lower serum levels, indicating that Sim was beneficial to liver function and blood lipid levels in HCC. In conclusion, these results show that Sora + Sim co-treatment could enhance the effect (sensitivity) of Sora on HCC by promoting apoptosis and suppressing glycolysis.

### Sim enhances Sora sensitivity by inhibiting PKM2, HIF-1α and PPAR-γ

**Fig. 5:**
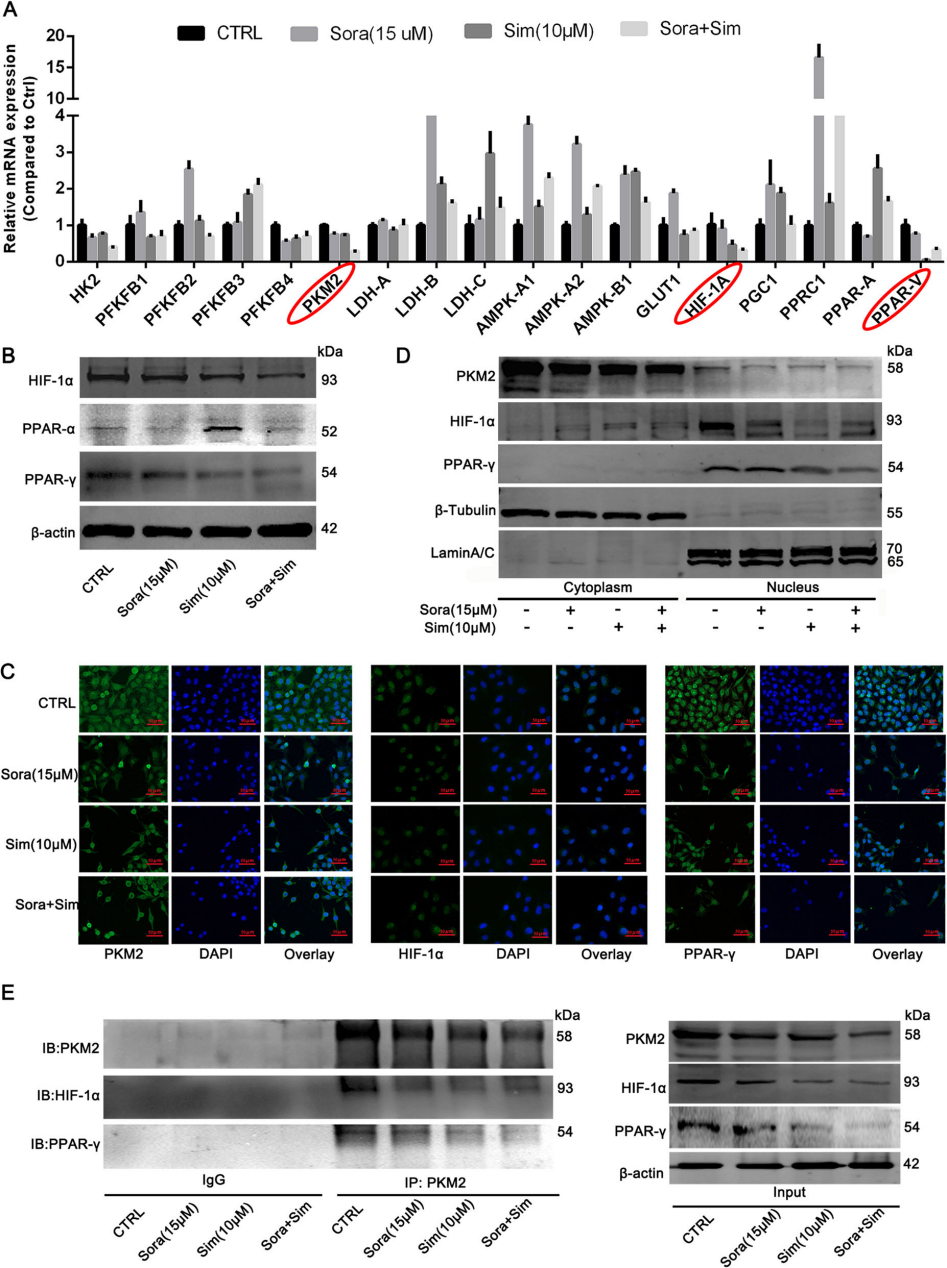
PKM2, HIF-1α and PPAR-γ may be involved in Sora and Sim co-treatment effects. (**a**) qPCR results of 18 genes associated with glycolysis, glucose metabolism and fatty acid metabolism. (**b**) Western blotting analysis of HIF-1α, PPAR-α and PPAR-γ in LM3-SR cells. (**c**) The IF staining of PKM2, HIF-1α and PPAR-γ in LM3-SR cells. (**d**) A cytoplasm-nuclear protein extraction kit used to analyze the distribution of PKM2, HIF-1α and PPAR-γ in LM3-SR cells. LaminA/C and β-tubulin were used as internal references for the nucleus and cytoplasm, respectively. (**e**) Co-IP assay used to determine the interaction between PKM2, HIF-1α and PPAR-γ

We next examined glycolysis, glucose and fatty acid metabolism-related genes by qPCR (Fig. 5a). Among 18 potential genes, we found that Sora + Sim co-treatment decreased the transcription of PKM2, hypoxia inducible factor-1α (HIF-1α) and peroxisome proliferator–activated receptor γ (PPAR-γ) the most. Western blotting analysis of PKM2, HIF-1α and PPAR-γ also showed a trend towards decreased protein levels in the Sora + Sim group. As seen in Fig. 5a, Sim activated the transcription of PPAR-α, the protein expression of PPAR-α was also detected by western blotting. However, the results seen in Fig. 5b show that the co-treatment with Sora and Sim did not inhibit PPAR-α expression to the same extent as Sora alone. Immunofluorescence staining further showed that the fluorescence intensity of PKM2, HIF-1α and PPAR-γ were reduced by Sora + Sim co-treatment (Fig. 5c). Moreover, from the localization of PKM2, we see that Sora + Sim co-treatment does not only inhibit the total expression of PKM2 (Fig. 5b-c), but also specifically inhibits its nuclear expression (Fig. 5c). These results were verified using a cytoplasm-nuclear protein extraction kit (Fig. 5d). Besides, the results in Fig. 5d showed that most of HIF-1α and PPAR-γ were localized in the nucleus, and Sim treatment can inhibit the expression of both HIF-1α and PPAR-γ in the nucleus. Given that PKM2, HIF-1α and PPAR-γ can localize to the nucleus, we conducted a Co-IP assay to determine their interaction. The results presented in Fig. 5e demonstrated that both HIF-1α and PPAR-γ can be pulled-down by PKM2, indicating that both HIF-1α and PPAR-γ can interact with PKM2 in the nucleus. Based on this finding, we concluded that Sim enhanced the sensitivity of Sora by inhibiting the expression and interaction of PKM2, HIF-1α and PPAR-γ.

### Sim enhances the sensitivity of Sora by down-regulating the HIF-1α/PPAR-γ/PKM2 axis

**Fig. 6:**
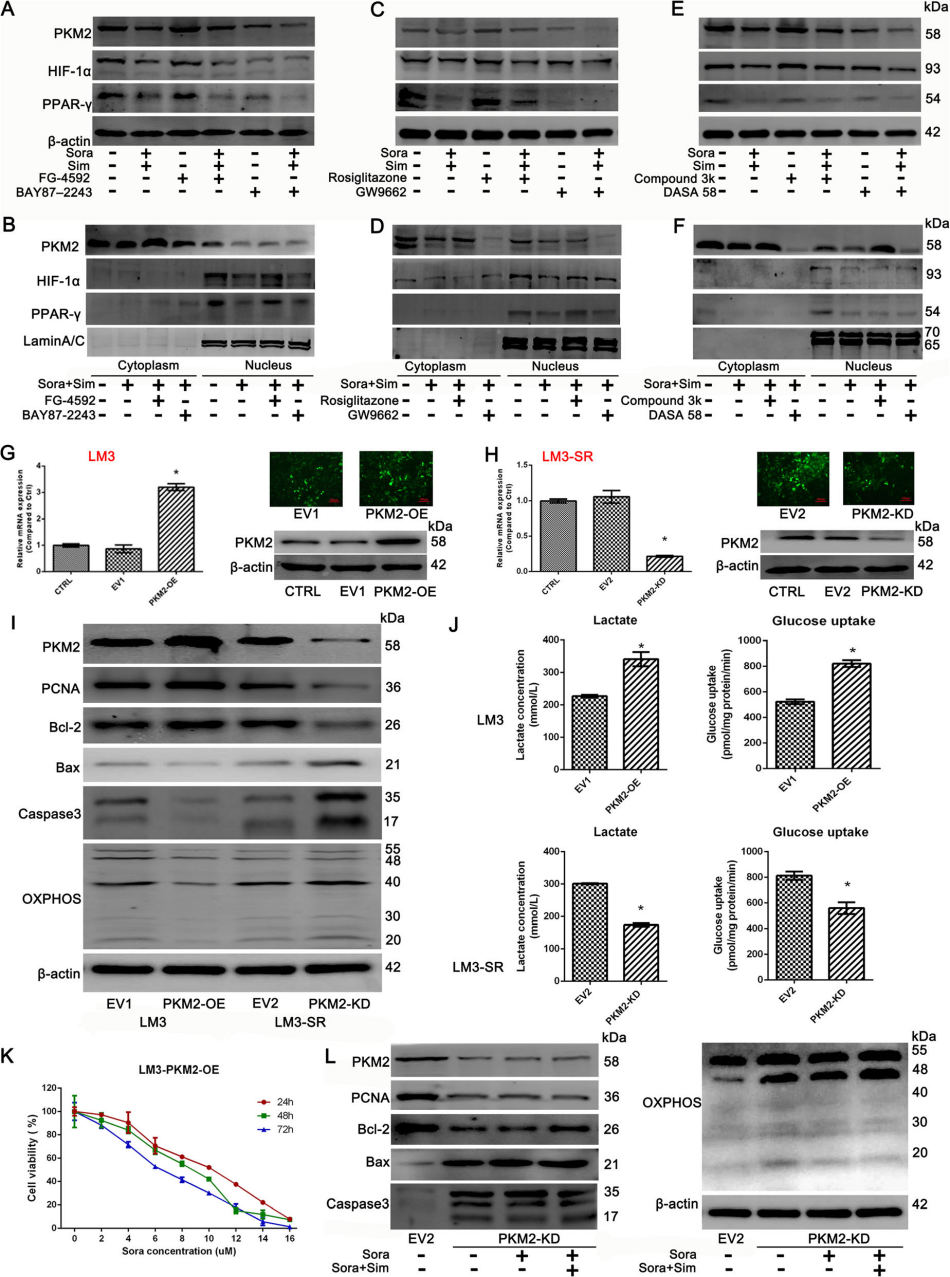
Sim enhanced the sensitivity of Sora by down-regulating the HIF-1α/PKM2 axis. (**a**) FG-4592, the activator of HIF-1α, and BAY87–2243, the inhibitor of HIF-1α were used to explore the role of HIF-1α on PPAR-γ and PKM2 by western blotting. (**b**) The analysis of nuclear expression of HIF-1α, PPAR-γ and PKM2 after treated with BAY87–2243 or FG-4592. (**c**) Rosiglitazone, the activator of PPAR-γ, and GW9662, the inhibitor of PPAR-γ were used to explore the role of PPAR-γ on HIF-1α and PKM2 by western blotting. (**d**) The analysis of nuclear expression of HIF-1α, PPAR-γ and PKM2 after treated with Rosiglitazone or GW9662. (**e**) Compound 3 k, the inhibitor of PKM2, and DASA 58, the activator of PKM2 were used to explore the role of PKM2 on HIF-1α and PPAR-γ by western blotting. (**f**) The analysis of nuclear expression of HIF-1α, PPAR-γ and PKM2 after treated with compound 3 k or DASA 58. (**g**) Verification of PKM2 over expression in LM3 cells via lentivirus transfection. (**h**) The verification of PKM2 knockdown in LM3-SR cells via lentivirus transfection. (**i**) Western blotting analysis of critical proteins in LM3 cells over expressing PKM2 or in LM3-SR cells with PKM2 knocked-down. (**j**) Glycolysis levels in LM3 cells over expressing PKM2 or in LM3-SR cells with PKM2 knocked-down. Results indicated by lactate production and glucose uptake levels. (**k**) CCK8 analysis of the effect of Sora in LM3-PKM2-OE cells at 24 h, 48 h and 72 h. (**l**) Effect of Sora and Sora + Sim co-treatment on LM3-SR-PKM2-KD cells. The data were represented as mean ± SD. * indicates *p* < 0.05 vs. CTRL or EV group

Activators and inhibitors of the HIF-1α/PPAR-γ/PKM2 axis were used to explore the involvement of HIF-1α, PPAR-γ and PKM2 on the effects seen with Sim. As shown in Fig. 6a, after FG-4592 treatment (50 μM) [35, 36], the expression of HIF-1α, PPAR-γ and PKM2 were all up-regulated; however, in the Sora + Sim group, the up-regulation after FG-4592 treatment was reversed. However, after application of the HIF-1α inhibitor BAY87–2243 (10 mM) [37], the expression of HIF-1α, PPAR-γ and PKM2 were all down-regulated. Furthermore, after co-treated with Sora + Sim, their inhibitory effect were overlaid. The nuclear expression of HIF-1α, PPAR-γ and PKM2 was also detected using a nuclear and cytoplasmic protein extraction kit again, and the results reflected that the nuclear expression of HIF-1α, PPAR-γ and PKM2 can be all inhibited by Sora + Sim co-treatment, and the changes of them after FG-4592 or BAY87–2243 treatment were similar to the total protein (Fig. 6b). These effects imply that HIF-1α is the up-stream regulator of PPAR-γ and PKM2.

Next, we used an activator and inhibitor of PPAR-γ for further confirmation. In Fig. 6c, after treatment with the PPAR-γ activator Rosiglitazone (10 μM) [38, 39], the expression of both PPAR-γ and PKM2 were increased, whereas the expression of HIF-1α remained similar to the CTRL group. If however, Rosiglitazone was co-treated with Sora + Sim, the increases seen in PPAR-γ and PKM2 were abolished, and the expression of HIF-1α remained the same as in the Sora + Sim group. Moreover, after treatment with the PPAR-γ inhibitor GW9662 (2 μM) [38, 39], the expression of PPAR-γ and PKM2 were decreased, whereas the expression of HIF-1α remained unchanged. Furthermore, when GW9662 was co-treated with Sora + Sim, the decrease in PPAR-γ and PKM2 was enhanced. As for the nuclear fractions, the changes of HIF-1α, PPAR-γ and PKM2 were similar to the total protein when treated with Rosiglitazone or GW9662 (Fig. 6d). These results suggest that HIF-1α was the up-stream regulator of PPAR-γ, and PKM2 was possibly the down-stream regulator of PPAR-γ.

Based on the above findings, we went on to use both an activator and inhibitor of PKM2 (Fig. 6e). Since PKM2 is a pyruvate kinase, the activator DASA 58 was able to elevate its enzymatic activity but inhibit its expression, and the inhibitor compound 3 K was able to reduce the enzymatic activity of PKM2 but promote its expression. Therefore after treatment with compound 3 K (3 μM) [40], the expression of PKM2 was up-regulated, whereas the expression of HIF-1α and PPAR-γ were found to be the same as the CTRL group. However, if compound 3 K was co-treated with Sora + Sim, the up-regulation of PKM2 was decreased slightly, and the expression of HIF-1α and PPAR-γ remained the same as the Sora + Sim group. The effect of co-treatment with DASA 58 (40 μM) was however, opposite to that seen with compound 3 K [29]. The analysis of nuclear fractions also showed that the changes of HIF-1α, PPAR-γ and PKM2 were similar to the total protein when treated with compound 3 K or DASA 58 (Fig. 6f). In conclusion, these results provide further evidence for an up-stream regulatory role for HIF-1α and PPAR-γ on PKM2. The HIF-1α/PPAR-γ/PKM2 axis was suppressed in Sim treatment.

PKM2 is not only a rate-limiting enzyme during glycolysis, but also a critical transcription factor in the nucleus. We next looked for a role for PKM2 in Sora resistance using lentiviral transfection. Figure 6g shows the over expression of PKM2 in LM3 cells, and Fig. 6h the knock down of PKM2 in LM2-SR cells. As revealed in Fig. 6i and j, if LM3 cells were overexpressed with PKM2, the expression of PCNA was elevated in LM3 cells, and the expression of Bax, Caspase 3 and OXPHOS were inhibited. Moreover, lactate production levels and glucose uptake levels were enhanced in the PKM2-OE group in LM3 cells. This implied that the over expression of PKM2 in LM3 cells may lead to resistance to Sora. This hypothesis was then verified by using the CCK8 assay, as seen in Fig. 6k. The results showed that the IC50 of LM3-PKM2-OE cells to Sora was 8.68 μM (LM3 cells were 4.47 μM) at 24 h. However, if PKM2 was knocked down in LM3-SR cells, the proliferation, inhibition of apoptosis and glycolysis levels were all suppressed, suggesting that the resistance to Sora in LM3-SR cells was recovered (Fig. 6i and j). The effect of Sora + Sim co-treatment in LM3-SR-PKM2-KD cells was also detected by western blotting (Fig. 6l). The results demonstrated that if PKM2 was knocked down in LM3-SR cells, Sim failed to inhibit the expression of PKM2, PCNA, Bcl-2, or increase Bax, Caspase 3 and OXPHOS in the Sora + Sim group when compared to the Sora group alone. Therefore, in summary, these results confirmed the involvement of the HIF-1α/PPAR-γ/PKM2 axis in Sim treatment, and proved that the over expression of PKM2 may lead to resistance to Sora in LM3 cells, whereas the knock down of PKM2 may improve Sora resistance in LM3-SR cells.

## Discussion

Sora resistance is the main limiting factor in the effective treatment of advanced HCC. In humans, the average time for Sora resistance to occur is approximately 12.2 moths but can vary from months to years [6]. The establishment of a Sora-resistant HCC cell line usually takes about 12 weeks. In our study, we successfully established LM3-SR cells, which could tolerate 10 μM Sora (with an IC50 of 16.33 μM) to study the relationship between Sora resistance and aerobic glycolysis in vivo and in vitro.

The mechanisms of Sora resistance remain obscure, but new insights include a higher EGFR, c-Jun and Akt activation in HCC cells, as well as increased EMT, cancer stem cells, hypoxic environment, autophagy, and exosomes [2, 5, 34, 41, 42]. Recently, several studies reported that aerobic glycolysis may also contribute to Sora resistance. In the 1920s, Otto Warburg found that even in conditions of sufficient oxygen levels, tumor cells prefer to metabolize glucose via glycolysis to lactate, rather than OXPHOS to generate metabolic intermediates rapidly, and this phenomenon is now termed the Warburg effect [43, 44]. Fiume et al. found that Sora treatment could damage OXPHOS and promote aerobic glycolysis in cells grown in a glucose rich environment [45]. Reyes et al. found that Sora and 2-Deoxyglucose (2-DG) co-treatment could synergistically inhibit the proliferation of Sora resistant HCC cells by inhibiting ATP production [34]. It was also shown by Wong et al. that 2-DG can reverse Sora resistance in HCC [44]. Therefore, in our study, we used the original LM3 and LM3-SR cells to detect glycolysis levels. Our results show that LM3-SR cells exhibited a higher lactate production and glucose uptake levels when compared to LM3 cells, which can support the enhanced aerobic glycolysis seen during Sora resistance in HCC cells.

Additionally, Sim, is a cholesterol-lowering agent, and recently has also been reported to participate in the suppression of glycolysis and Sora resistance. Christie et al. found that statins can partially block the utilization of glycolytic ATP [46]. Huang et al. found that the combination of pitavastatin and paclitaxel can significantly decrease the glycolytic rate in renal carcinoma [47]. Nowis et al. reported that statins impaired glucose uptake in human cells derived from the liver [48]. These findings suggest that Sim may also be effective in inhibiting glycolysis and may be a potential agent to help improve glycolysis-mediated Sora resistance. In our study therefore, based on the pluripotent nature of statins, we wanted to combine Sim with Sora to explore whether Sim can improve the sensitivity of LM3-SR cells to Sora. Firstly, we detected the effect of Sim on LM3 and LM3-SR cells alone. We found that LM3-SR cells were more sensitive to Sim (10 μM) than LM3 cells, which was reflected by a higher inhibition of proliferation rate and greater apoptosis. In our study, Sim (10 μM) was also effective at inhibiting aerobic levels by reducing glycolytic lactate and glucose production, and inhibiting the expression of glycolysis related protein expression. Secondly, combined treatments using both Sora and Sim were performed, and the results showed that Sim synergistically enhanced the sensitivity of LM3-SR cells to Sora in a combined treatment, as reflected by a CI value less than 1. Increased inhibition of proliferation and higher apoptosis were also seen, both in vivo and in vitro.

The mechanism underlying the re-sensitizing effect of Sim on Sora was explored next. There are three rate-limiting enzymes during aerobic glycolysis: HK2, PFK1 and PKM2. Among them, PKM2, can catalyze the last step of glycolysis, and is up-regulated in many tumors [49, 50]. PKM2 has three functions in cancer cells: (1) cytoplasmic PKM2 is a tetramer with high enzyme activity, and takes part in glycolysis to provide increased metabolic intermediates for cancer cell biosynthesis, (2) the dimeric isoform of PKM2 can translocate to nucleus and act as a transcriptional co-activator, thereby facilitating the transcription of genes beneficial to growth, such as GLUTs, HIF-1α, VEGF-A, Bax, Bcl-2, and PCNA. (3) PKM2 can also translocate to the mitochondria under oxidative stress to interact with Bcl-2/Bcl-xl, causing the inhibition of cancer cell apoptosis [51–53]. Based on the critical role of PKM2, Zhang et al. reported that silencing PKM2 could re-sensitize Hep3B-SR and LM3-SR cells to Sora [33]. Wong et al. also found that the PRMT6-ERK-PKM2 regulatory axis takes part in Sora resistance and glucose metabolism in HCC [44]. In our study, we firstly found that Sora + Sim co-treatment can not only inhibit the expression of PKM2, but also inhibit the nuclear translocation of PKM2 by IF staining. Secondly, we revealed that the over expression of PKM2 in LM3 cells led to Sora resistance. However, knock down of PKM2 in LM3-SR cells effectively restored the sensitivity of LM3-SR cells to Sora, and Sim + Sora co-treatment failed to inhibit the proliferation or increase apoptosis in LM3-SR-PKM2-KD cells. These finding provide further proof for a critical role of PKM2 in the synergic co-treatment of Sora and Sim.

**Fig. 7:**
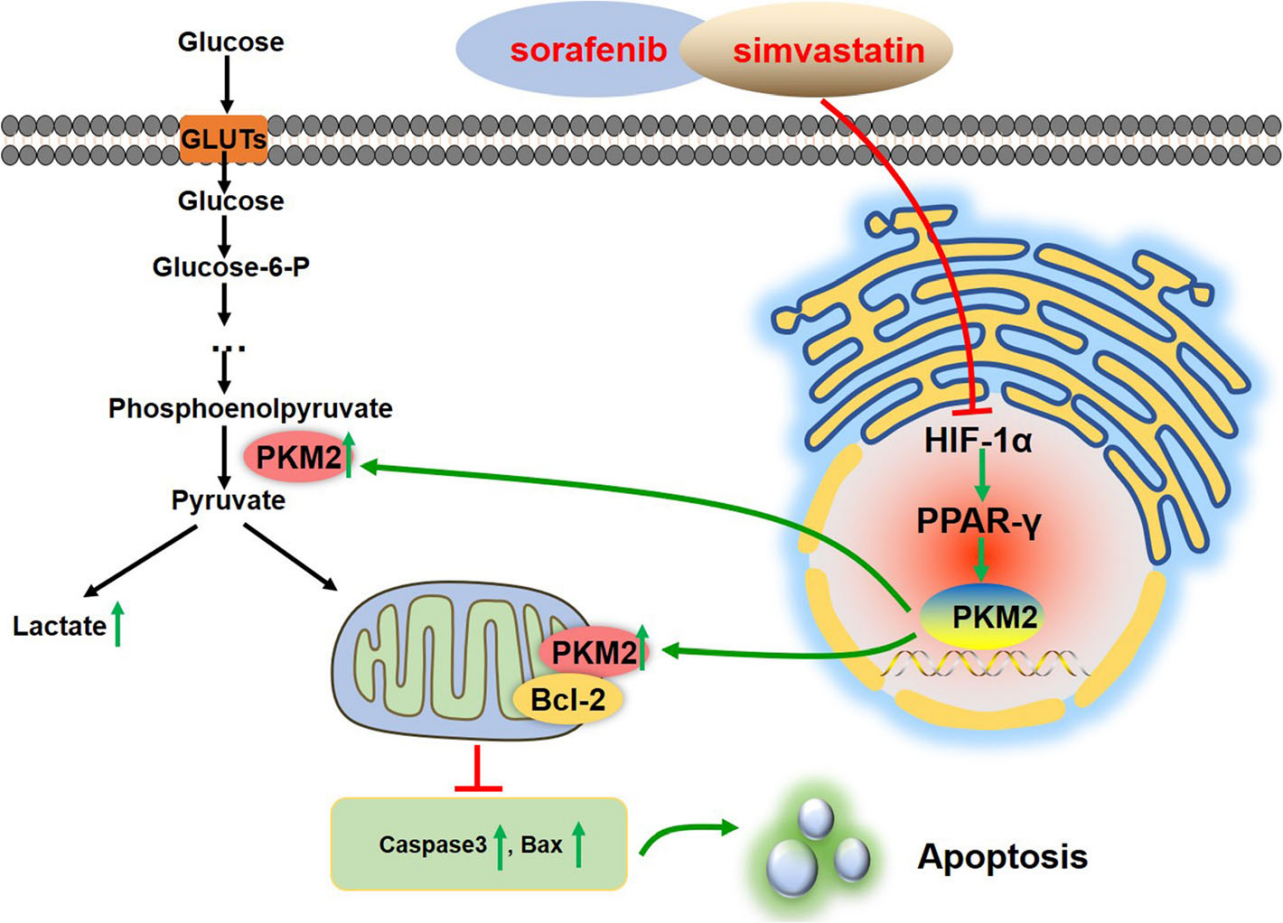
The mechanism of Sora and Sim co-treatment in LM3-SR cells. Sim can enhance the sensitivity of LM3-SR cells to Sora by suppressing the HIF-1α/PPAR-γ/PKM2 axis, leading to the down-regulation of PKM2 expression in the nucleus and cytoplasm, thereby inhibiting glycolysis, proliferation and promote apoptosis in LM3-SR cells

The expression of the PKM2 gene can also be driven by various factors, including HIF-1α, STAT3, β-Catenin, and NF-κB [54–56]. In this study we found that both HIF-1α and PPAR-γ may be involved in the re-sensitization effect of Sim on Sora resistance. HIF-1α, which is a regulatory factor involved in the cellular response to hypoxia, can promote glycolysis in cancer cells via the direct transcriptional activation of glycolysis related genes, including glucose transporters (GLUTs) and PKM2 [57]. PPAR-γ plays an important role in maintaining energy homeostasis through the modulation of glucose and lipid metabolism, and PPAR-γ is usually overexpressed in cancer cells causing accelerated tumor growth [58, 59]. However, the role of PPAR-γ agonists and antagonists in tumor treatment is complex, as both have been found to inhibit the growth of tumor cells [58, 60–62]. PPAR-γ has been found to promote glucose uptake during lipid metabolism, and can induce the expression of glycolytic proteins, including GLUT-4 [63]. It has been seen that atorvastatin can inhibit the HIF-1α/PPAR-γ pathway and inhibit the survival of induced pluripotent stem cells [15]. In addition, Panasyuk et al. also reported that PPAR-γ can promote the expression of PKM2 and HK2 in fatty liver [59]. In our study, we found that PKM2 can interact with both HIF-1α and PPAR-γ using CO-IP assay. We subsequently used activators and inhibitors of HIF-1α, PPAR-γ and PKM2 in LM3-SR cells, and the results confirmed a role for the HIF-1α/PAR-γ/PKM2 axis in LM3-SR cells. In addition, our rescue experiments showed that the co-treatment effects of Sora + Sim can be reversed by the HIF-1α, and PPAR-γ activators FG4592 and Rosiglitazone and the PKM2 inhibitor compound 3 k. These findings provide convincing evidence for the HIF-1α/PAR-γ/PKM2 axis as the target for Sim during re-sensitization of HCC cells to Sora (Fig. 7).

The mechanism underlying Sora resistance is complex. Except for the HIF-1α/PPAR-γ/PKM2 axis investigated in the present study, the PKM2 isoform, PKM1, is also reported to increase glycolysis through autophagy and cause cancer chemoresistance [64, 65]. Moreover, the oncogene Myc, which includes c-Myc, N-Myc, and L-Myc, plays an important role in aerobic glycolysis in HCC as well. C-Myc is reported to overexpressed in HCC, and can promote the Warburg effect by increasing the expression of glycolytic related markers, such as GLUT1, LDH and PKM2 [66–69]. Conversely, c-Myc can also be regulated by PKM2 in the nucleus, for PKM2 can translocate into nucleus and act as a coactivator of β-catenin to induce c-Myc expression, leading to the expression of c-Myc targeted genes [30, 67]. In addition, high levels of c-Myc activity will enhance the PKM2/PKM1 ratios [49, 70]. c-Myc also increases the glutaminolysis in cancer cells and then promotes the progression of cancer [71]. Based on these, we proposed that c-Myc may be a possible for the treatment of Sora resistance in HCC, and this can be investigated in the future.

## Conclusions

In conclusion, our study investigated the promising role of Sim in improving HCC resistance to Sora, and we found that: (1) Sim was safe for co-treatment with Sora in vivo and did not aggravate liver function or organ damage. (2) Sim can inhibit the HIF-1α/PAR-γ/PKM2 axis, causing the suppression of PKM2-mediated glycolysis, decrease proliferation and increased apoptosis in HCC cells, thereby re-sensitize HCC cells to Sora.

## Supplementary information

**Additional file 1 : Figure S1.** The effect of Sora and Sim co-treatment on the naïve Sora-resistant Bel-7402 cells. (A) The glucose uptake and lactate production levels in LM3, LM3-SR and Bel-7402 cells. (2) The glycolytic enzymes and OXPHOS expression in LM3, LM3-SR and Bel-7402 cells. (C) The effect of Sim on Bel-7402 cell viability using CCK8 assay. The IC50 of Sim on Bel-7402 cells was 22.73 μM. (D) The effect of Sora(8 μM) and Sim (10 μM) co-treatment on Bel-7402 cells, by detecting the proliferation, apoptosis and glycolysis related markers using western blotting.

**Additional file 2 : Figure S2.** The effects of Sora + Sim co-treatment on LM3 cells. (A) The combined treatment analysis of Sora and Sim on LM3 cells using Calcusyn. The dose-effect curve, Fa-CI plot and Fa-DRI plots are shown. Sora (5 μM) and Sim (10 μM) resulted in CI value of 0.802, and the DRI for Sora was 1.323, revealing a synergic effect. (B) Flow cytometry analysis of the effect of Sora and Sim co-treatment in LM3 cells. (C) Glycolysis levels of Sora and Sim co-treatment in LM3 cells, reflected by lactate production and glucose uptake levels. (D) Western blotting analysis of critical proteins.

## Data Availability

The datasets used and/or analysed during the current study are available from the corresponding author on reasonable request.

## Abbreviations

2-DG: 2-Deoxyglucose
CCK-8: Cell counting kit
CI: Combination index
Co-IP: Co-immunoprecipitation
CTRL: Control
DRI: Dose reduction index
EGFR: Epidermal growth factor receptor
EMT: Epithelial-mesenchymal transition
Fa-CI: Fraction affected-combination index
GLUT: Glucose transporter
H&E: Hematoxylin and eosin
HCC: Hepatocellular carcinoma
HDL-C: High-density lipoprotein cholesterol
HIF-1α: Hypoxia inducible factor-1α
HK2: Hexokinase 2
HMG CoA: Hydroxymethylglutaryl coenzyme A
IHC: Immunohistochemistry
LDL-C: Low-density lipoprotein cholesterol
LM3-SR: Sora-resistant LM3 cell line
OXPHOS: Oxidative phosphorylation
PFK1: Phosphofructokinase 1
PKM2: Pyruvate kinase, type M2
PPAR-γ: Peroxisome proliferator–activated receptor γ
qRT-PCR: Quantitative reverse transcription-polymerase chain reaction
SD: Standard deviation
Sim: Simvastatin
Sora: Sorafenib
TCHO: Total cholesterol
TG: Triglyceride
TUNEL: Terminal deoxynucleotidyl transferase dUTP nick end labeling
