# Supplementary figures and images for: Simvastatin re-sensitizes hepatocellular carcinoma cells to sorafenib by inhibiting HIF-1α/PPAR-γ/PKM2-mediated glycolysis
Source: J Exp Clin Cancer Res. 2020 Jan 30;39:24. doi: 10.1186/s13046-020-1528-x (PMC6993409; doi:10.1186/s13046-020-1528-x)

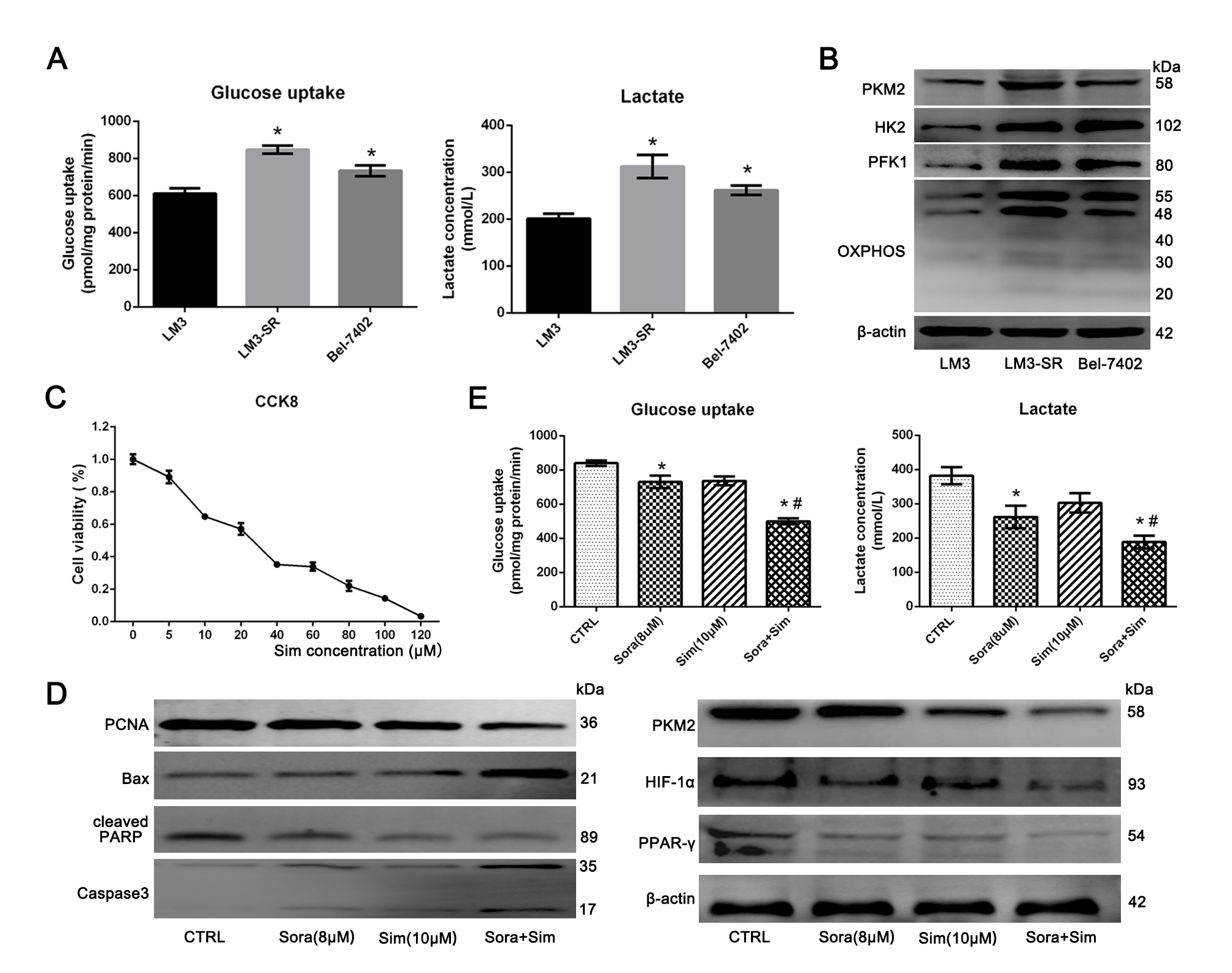

Supplement: Supplementary file 1 — Additional file 1 : Figure S1. The effect of Sora and Sim co-treatment on the naïve Sora-resistant Bel-7402 cells. (A) The glucose uptake and lactate production levels in LM3, LM3-SR and Bel-7402 cells. (2) The glycolytic enzymes and OXPHOS expression in LM3, LM3-SR and Bel-7402 cells. (C) The effect of Sim on Bel-7402 cell viability using CCK8 assay. The IC50 of Sim on Bel-7402 cells was 22.73 μM. (D) The effect of Sora(8 μM) and Sim (10 μM) co-treatment on Bel-7402 cells, by detecting the proliferation, apoptosis and glycolysis related markers using western blotting. [file 13046_2020_1528_MOESM1_ESM.jpg]

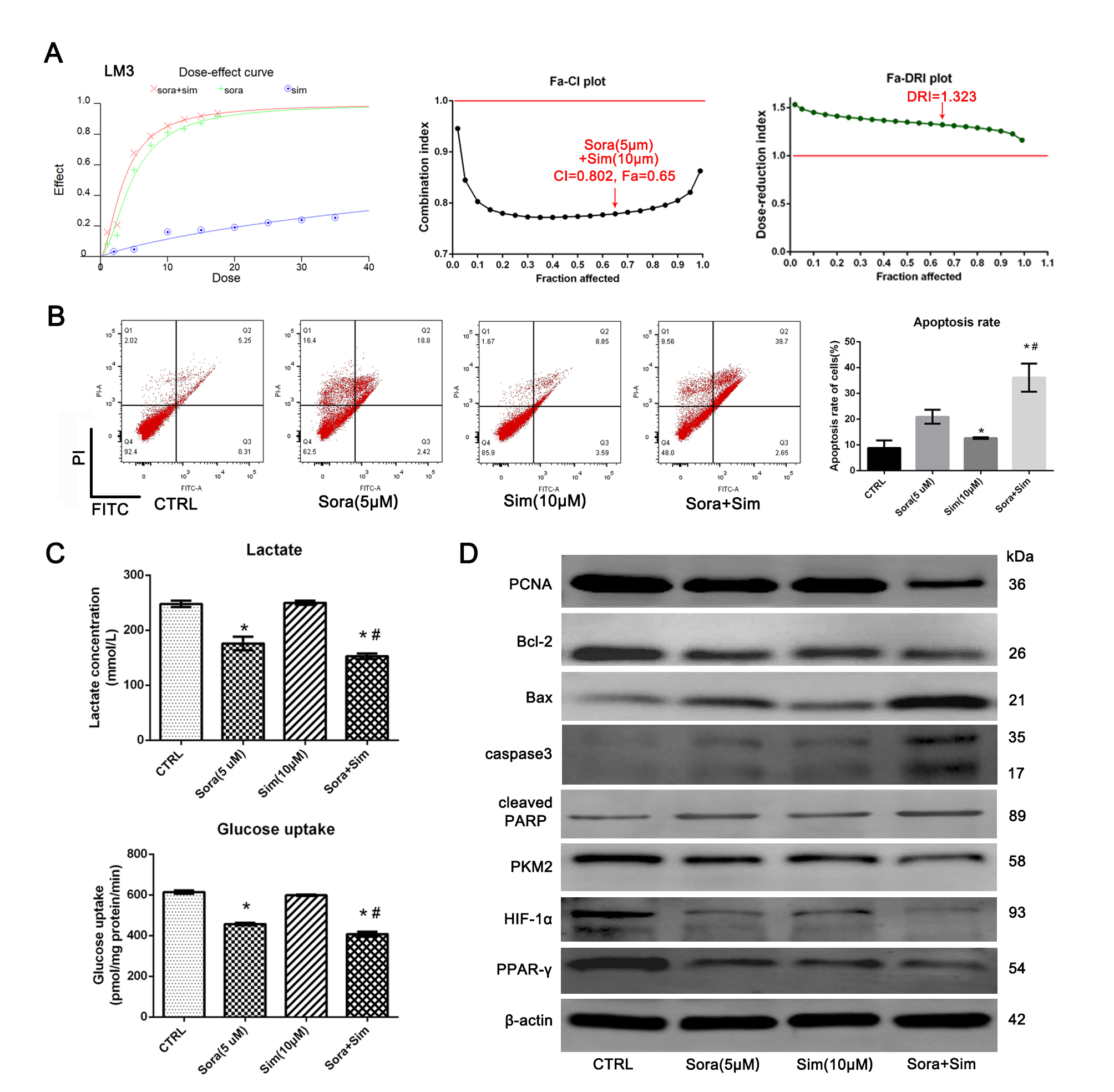

Supplement: Supplementary file 2 — Additional file 2 : Figure S2. The effects of Sora + Sim co-treatment on LM3 cells. (A) The combined treatment analysis of Sora and Sim on LM3 cells using Calcusyn. The dose-effect curve, Fa-CI plot and Fa-DRI plots are shown. Sora (5 μM) and Sim (10 μM) resulted in CI value of 0.802, and the DRI for Sora was 1.323, revealing a synergic effect. (B) Flow cytometry analysis of the effect of Sora and Sim co-treatment in LM3 cells. (C) Glycolysis levels of Sora and Sim co-treatment in LM3 cells, reflected by lactate production and glucose uptake levels. (D) Western blotting analysis of critical proteins. [file 13046_2020_1528_MOESM2_ESM.jpg]
